# Legal & ethical compliance when sharing biospecimen

**DOI:** 10.1093/bfgp/elx008

**Published:** 2017-04-27

**Authors:** Tomas Klingstrom, Erik Bongcam-Rudloff, Jane Reichel

**Keywords:** ethics, biobank, sample access, genomics, DNA

## Abstract

When obtaining samples from biobanks, resolving ethical and legal concerns is a time-consuming task where researchers need to balance the needs of privacy, trust and scientific progress. The Biobanking and Biomolecular Resources Research Infrastructure-Large Prospective Cohorts project has resolved numerous such issues through intense communication between involved researchers and experts in its mission to unite large prospective study sets in Europe. To facilitate efficient communication, it is useful for nonexperts to have an at least basic understanding of the regulatory system for managing biological samples.

Laws regulating research oversight are based on national law and normally share core principles founded on international charters. In interview studies among donors, chief concerns are privacy, efficient sample utilization and access to information generated from their samples. Despite a lack of clear evidence regarding which concern takes precedence, scientific as well as public discourse has largely focused on privacy concerns and the right of donors to control the usage of their samples.

It is therefore important to proactively deal with ethical and legal issues to avoid complications that delay or prevent samples from being accessed. To help biobank professionals avoid making unnecessary mistakes, we have developed this basic primer covering the relationship between ethics and law, the concept of informed consent and considerations for returning findings to donors.

## Introduction

The Biobanking and Biomolecular Resources Research Infrastructure-Large Prospective Cohorts project has provided valuable experience on the issues of sample access through its open calls to provide funding for accessing biobanked samples. For the cohorts who did participate in various projects, attaining ethics approval and sorting the legal issues were time-consuming but not insurmountable tasks because of intense communication between involved researchers and experts.

The risk of biobank samples being used in an inappropriate manner has received increasing attention in scientific discourse. In comparison, the threat of under-utilization of samples or an inability to return the benefits of research to donors has received relatively little attention, despite also being among the chief concerns of interviewed donors [1]. Furthermore, the genomic revolution means that pretty much any sample can be considered to contain potentially identifiable personal data in the form of DNA. Researchers therefore face an intricate extra-legal regulatory system complete with steering documents (ethics guidelines), overseeing bodies (research ethics committees) and formal procedures (informed consent) [2] when attempting to access samples.

Although laws regulating research oversight have been implemented differently in every country, there is a similarity of core principles founded on international charters such as the Helsinki Declaration. International consortia have translated these core principles into policies, procedures, tools and governance that facilitate interoperability between biobanks across national borders in a manner acceptable to national law makers [3–5], thereby enabling the scientific community to operate despite a lack of clarity and international agreements that may provide a stable and enabling environment for international collaboration [6, 7].

As biobanks mature, priorities tend to shift [8], and it is not uncommon that biobanks find themselves prevented from providing samples due inappropriate decisions taken several years earlier. These complications are often the result of requests with unforeseen requirements causing uncertainties if given consents are sufficient and how or if information from new research projects should be returned to the donors. The primer therefore covers how these obligations are governed under international agreements and national law, the practice of establishing this relationship by the concept of informed consent and the difficulties on deciding when and what information should be provided to sample donors.

## Hard and soft law, the key to international collaboration

The national legal framework of biobanking is often substantially different even between countries of comparable jurisdictional systems [9]. To accommodate international collaboration, it is therefore necessary to rely on ‘soft law’ or extra-legal means to bridge the gap between the national legal systems, which operate on a ‘one nation, one law, one project’ approach [10].

When dealing with such matters, it is therefore important to understand and recognize how research is regulated by a combination of ‘hard law’ and ‘soft law’ where the terms can be defined as follows:

**Hard law**: Binding legal instruments, either in the form of international law (conventions, treaties or agreements) or national law (statutory law). International law is often drafted in a more general form and subsequently implemented in national law. For the individual researcher, it is most often the national statutory law that regulates the legality of actions.

**Soft law:** Nonbinding instruments such as guidelines and codes of conducts that may lay down suitable and commonly accepted ways to deal with a matter. Soft law in different forms varies in form from openly phrased to rather strictly defined rules, bearing close resemblance to hard law.

Hard law is codified in legal text, which makes it relatively straightforward for a trained expert to access and identify the relevant laws. Soft law is on the other hand more flexible but makes it harder to find and understand the regulatory mechanisms, as it allows governmental and nongovernmental experts to update regulations and standards without requiring active engagement of law-making bodies, and often these experts may be specified in hard law as bodies tasked with providing legally binding regulations and decisions. Funding bodies are becoming an increasingly important source of soft law by enforcing contracts requiring certain guidelines or procedures to be followed by researchers to be eligible for funding.

For European researchers, an important source of this kind of regulation is the European Union (EU) funding programs managed by the European Commission. It requires applicants to state in their proposal that they will conform to specific standards [11] where failure to comply mean that the researcher will not be eligible to receive the funds provided by the grant.

Similar approaches are not only used for international projects but are also a way for national agencies to harmonize activities in nations where legislation is done at a regional or state level. For example, in the United States, the National Research Council stipulates the following for the international transfer of embryonic stem cells:

If a U.S.-based investigator collaborates with an investigator in another country, the ESCRO committee may determine that the procedures prescribed by the foreign institution afford protections consistent with these guidelines, and the ESCRO committee may approve the substitution of some of or all of the foreign procedures for its own. [12]

These guidelines are defined by one selected group of experts (the National Research Council) who delegate decisions to another group of experts [the Embryonic Stem Cell Research Oversight (ESCRO) Committee], which is charged with deciding if there is a comparable set of checks and balances in the partner country in the form of a, yet to be identified, third group of experts. These guidelines are a good example of how a soft law approach with several layers reduces transparency in return for increased flexibility, as guidelines, review committees and research practitioners make up an ever-changing system of stakeholders. Under such circumstances, collaboration is substantially more likely to be accepted between nations where the respective authorities have had the possibility to become familiar with each other’s customs and traditions, and above all, where the legal requirements applicable to the matter have been enacted as a result of international agreements. A lack of trust, harmonization or the local preferences of the committee may therefore significantly affect the outcome of an application for the transfer of data or samples. Decisions by judicial authorities covering one of the partners in a collaboration may also have an immediate impact on international collaboration, as certain procedures are deemed to be in conflict with national law. The EU has, for example, chosen a high standard for data protection, as seen in the recent Safe Harbor-ruling from the Court of Justice of the European Union (C-362/14), where the US level of protection was found not to uphold an adequate protection.

However, most modern national laws are based on an ambition to adhere to a common set of core principles derived from the declaration of human rights and international declarations such as the Declaration of Helsinki [13]. This means that even if there is yet little legal harmonization between countries. There is a strong case for researchers to argue that institutional review boards should take into account decisions from review boards in other countries, in a soft version of a principle of mutual recognition.

## Consent as the basis of international collaboration

The signed consent form provides a receipt that verify that the donor has been provided with sufficient information to make an informed consent when donating his or her samples. Modern regulations regarding informed consent were codified in an international setting by the Helsinki declaration and Nuremberg code [14] as a result of the horrors in World War II and subsequent development. Respect for the autonomy of research subjects and their right to refuse participation in research does however have a much longer history in research [15] even if modern researchers may find certain practices troubling or even barbaric. For example, in the mid-19th century in America, it was considered acceptable for a slave owner to obtain consent for invasive experimental surgery from slaves [16]. While it for a modern person is hard, if not impossible to accept slavery or the concept of ‘a consenting slave’. From an academic context, this intuitive protest can be interpreted as an example of how we instinctively respect that a person in a position of dependence cannot make a truly autonomous decision [17]. The concept of donors as autonomous agents is one of the key concepts of modern research, and the question of identifying what information and freedom is necessary before a person can make an autonomous decision is therefore central to all forms of biobanking and genomic research with human participants.

When establishing a new biobank, it is important to rely on forward-looking consent procedures to ensure the future viability of the sample collection. A large number of different forms of consent have been proposed in scientific literature. But in practice, consent forms likely available to a biobank would need to result in a presumed, broad or specific kind of consent (Table 1). In bioethicist literature, concepts such as ‘tiered’ or ‘dynamic’ consent are suggested as compromises between specific or broad forms of consent. In practice, these forms of consent can either be broad or specific depending on whether the components of the consent are widely or narrowly specified. It is however not always possible or feasible to obtain information from a known, informed and willing donor. In some cases, a presumed consent is necessary, and several ethicists also argue that a consent can never be truly informed unless strict requirements are met [18–20].

**Table 1.** Forms of consent described in literature

Generalized category	Type of consent	Definition	Authors	Disagreement
No consent given	Presumed	Consent is presumed to have been given by donors to use their samples and information for all research unless they actively choose to opt out	Master *et. al*. and Hofman	
	Passive/tacit/silent consent	Presuming that the persons object if they do not consent	Hofman	
	Hypothetical consent	Consent under the presumption that a person would have consented to the treatment or research were she or he able to consent	Hofman	
A broad or specific consent	Future/deferred consent	Postponing the consent procedure	Hofman	
An extremely broad consent	General/blanket/open consent	Donors can actively consent once for the current study and all future research involving the general use of their samples and information	Master *et. al*., Hofman and Salvaterra *et al.*	Salvaterra refer to this as broad consent
May be either broad or specific depending on how the consent is formulated and the definition used by the reviewers	Broad	Donors can actively consent once for the current study and all future research within a broad field, e.g. cancer, diabetes or heart disease	Master *et. al*., Hofman and Salvaterra *et al.*	Salvaterra refer to this as partially restricted consent
	Delegated trustee	Donors can transfer consent to a trustee who is at arm’s distance from the biobank and consents on behalf of donors	Master *et al.*	
	Third-party oversight	Donors can actively consent to a general, broad or other model, but an ethics board must approve the study before the commencement of research using stored samples and information. This approach is emerging as a common component of biobanking governance schemes	Master *et al.*	
	Tiered	Donors can actively consent once for the current study and choose one or more broad fields of research or other options, i.e. whether they would be willing to have their samples used in research that result in commercialization. Other terms: line item or multilayered consent	Master *et al.*	
	Re-consent	Donors are informed and are required to consent to the current study and to each future research study involving the use of their samples and information	Master *et al.*	
	Specific informed consent	Allows the use of biological specimens and related data only in immediate research; forbids any future study that is not foreseen at the time of the original consent	Salvaterra *et al.*	

*Note*: Terms used in literature are not always univocal and may also be used with different levels of specificity. In the table, the specific definitions described by the authors have been clustered into more general of consent described in accordance with this article. The more specific definitions are listed in the column ‘Definition’, and the terms used to name them are outlined in ‘Type of consent’ and ‘Disagreement’.

When looking at large biobank infrastructures, a broad consent is favored among the major infrastructures [21–23] even if there still is debate among ethicists on how broad a consent can be while still maintaining the autonomy of the donor [24]. The dominance of broad consent in infrastructures based on soft law is in this context a good example of how soft law solutions allow society to adapt more quickly to new possibilities and risks compared with hard law where important laws may be debated for years before implementation [7].

Specific consent is by its nature reactive, as it is impossible to request specific consent for purposes not yet foreseen. As a response to this issue, proponents of specific consent have made numerous proposals where modern communication technology makes it possible to repeatedly (or dynamically) ask donors for consent [25]. Thus, initial consent only needs to cover foreseeable research, while new projects are made possible by a renewed consent, thereby, in the opinion of its proponents, creating a balance between maximizing the value of samples and the necessary safeguards to ensure that consent is truly informed.

However, research rarely takes place in clearly defined modules, and there is often a continuum where it is hard to define the acceptable threshold for clarity, which requires new consent [26]. In practice, this means that a biobank will require a similar independent ethics review board, regardless of if the biobank operates under a legislation requiring specific, broad or any other form of consent.

Recent research further underlines the support for a broad consent among biobank experts [20], but even a broad consent is limited in how much freedom may be given to researchers to initiate new projects. That an administrative framework remains in place for the sample collection and that the new research does not change the overall aims or governance structure are core conditions and may be regarded as a minimal set of regulations for a broad consent to remain valid [27]. For European needs, Carlo Petrini at the Bioethics unit of the Presidents office in Italy has conducted a bibliographical study of the requirements necessary to operate a biobank under a broad consent in Europe [28], suggesting that the following requirements must be met:

- Adequate sample coding procedures are used.
- Adequate procedures for personal data protection are used.
- The importance of the research aim is sufficient to justify conducting the study and is evaluated on a case-by-case basis by an ethics committee.
- The sensitivity of the data is evaluated on a case-by-case basis. Genetic information varies in sensitivity based on its significance, ranging from stringent protection to a lesser degree of protection.
- Generic research results are always released without specifically identification of individual subjects.
- ‘Opt-out’ consent is allowed for subsequent or secondary studies. Every subject must be guaranteed the possibility of withdrawing consent at any time.
- Participants must have adequate means of involvement, such as encouraging participant consultation or communicating information through the mass media before project initiation. The multiple modes of involvement should be complementary as opposed to mutually exclusive. It is especially important that forms of direct participation also be available, for example, by having population representatives serve on the ethics committees that will decide on the approval of the research before it begins.
- Measures to ensure transparency and supervision must be in place. Adequate supervisory, procedural and technical systems are necessary to guarantee information protection. Further, it is highly advisable to have external and independent supervisory bodies monitoring procedural correctness.

## The reporting of planned or incidental findings

Another controversial subject with far-reaching consequences for sample availability is whether researchers should be obliged to return information on findings to the donor [29]. There is currently no overall consensus on when to tell and when not to tell participants of incidental findings [30]. Careful planning of procedures to satisfy local or national expectations is therefore necessary to ensure that donor interests are managed properly. In cases where a study is based on samples, not yet collected, researchers can, and should, plan ahead to ensure that donors are properly informed at the time of consent on reporting procedures. For studies on samples already collected or where clinically relevant findings are incidental in nature, it instead becomes necessary for study manager to base their reporting procedures on their own judgment or guidelines provided by local experts or governing boards suited to the task.

**Figure 1.**
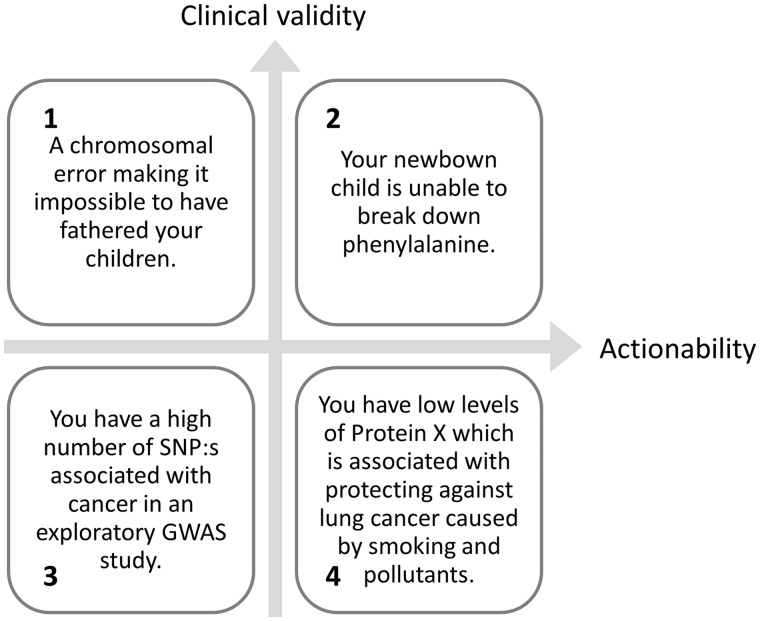
A breakdown of potential situations encountered when conducting genetic analysis on collected samples and practical examples of cases clearly belonging to each quarter. Support for returning information to the donor is strong when a finding is both reliable (possessing a high level of clinical validity) and actionable (the donor can act on the given information) as in the example given in Square 2 and, there is little support for providing information that is neither reliable nor actionable (Square 3). Decisions are harder and in greater need of consideration when the reliability of findings is low (Square 4) or when there is little the donor can do about the situation (Square 1).

Based on the conflicting opinions described by researchers conducting systematic reviews of the field, it would be foolhardy to claim that practitioners and ethicists are anywhere near a consensus in the field [21–23, 29]. It may however be possible to break down disclosure into two dimensions to separate situations where researchers are closer to consensus from areas where there still is severe disagreement (Figure 1).

Given this four-field breakdown and preceding information, ethicists are at least approaching a consensus on the lower left and upper right corners. Which mean that incidental findings with a high level of actionability and clinical validity should, if possible, be reported back to the donor [31] and findings of low validity and actionability should not be reported to the donors (2, upper right corner) ahead of [31] and (3, lower left corner). There is however no consensus on whether it is a moral necessity to actively look for such genes in genetic data, and many researchers also feel uncertain when judging if specific markers are actionable and clinically valid [31]. To support clinicians, the American College of Medical Genetics has taken initiatives to support researchers to reduce these difficulties with lists of valid and actionable genetic biomarkers [32], which can be consulted by clinicians to determine if incidental findings should be reported. The procedures for how and if findings are to be reported to the donor should be outlined to the donor at least by the time of consent, thereby helping to set donor expectations and define their future relationship with their donated samples.

This means that the researchers, when developing the consent form, must take care to ensure the long-term viability of the biobank and balance their obligations to donors with the scientific needs of the project. A high level of reciprocity cannot, for example, be offered in a biobank where a large portion of the research is expected to be conducted by external researchers limited to anonymized data to maintain privacy. It is therefore necessary that researchers make important decisions such as coding [33] versus anonymization before contacting potential donors for consent. Failure to do so may otherwise result in major issues in the future, as national laws on privacy or obligations outlined in the consent form may prevent the efficient usage of biospecimen.

## Concluding remarks

International collaboration relies on soft law connecting national legal systems, which creates an environment that is inconsistent, unfair and often lacking in transparency. But replacing the soft law with hard law may be even worse, as a codification of overly restrictive standards into law may stifle or outright halt scientific progress in regions within the jurisdiction of such laws [7]. Furthermore, it is unlikely that hard law solutions would be able to possess the necessary flexibility to keep up the pace with the rapid advancement of research and genomics.

As a researcher, it is easy to become frustrated and avoid engaging in such a complex, and ever-changing field of work. But despite calls for harmonization, it is unlikely that issues will be solved in the immediate future. There are significantly different legal traditions [34–37] as well as variation in public perception [38, 39] of research. Taken together, this makes it a perhaps insurmountable task to reach harmonization of national laws regarding biological samples and data protection. The legal obligations of biobank professionals concerning consent and reciprocity are therefore likely to change over time and remain areas associated with a high risk of interfering with the individual goals and aims of researchers.

In this context, adhering to best practices contributes to the long-term value of samples, as new implementations of soft law instruments and codified law are likely to take established best practices in consideration. Guidance and templates provided by international organizations such as International Society for Biological and Environmental Repositories (ISBER, www.isber.org), Global Alliance for Genomics and Health (http://genomicsandhealth.org), the Asian Network of Research Resource Centers (http://anrrc.org), the Biobanking and BioMolecular resources Research Infrastructure-European Research Infrastructure Consortium (www.bbmri-eric.eu) and the Human Heredity and Health in Africa (http://h3africa.org), here, form a platform for harmonization as well as generating the opportunities to build the mutual trust necessary to enable the transfer of samples or data. The role and function of these soft law tools must however take into account the constitutional aspect of the bioethical framework involving several human rights. Traditionally, these rights, and especially the limiting of the rights, are usually thought to be best regulated by democratically elected parliaments [40]. These international soft law tools do thus not supersede national authorities and courts, but their status as internationally recognized authorities may provide considerable support in achieving approval from institutional review boards acting under mandate from national laws.

It is therefore in the best interest of researchers to respect and promote core principles codified by international conventions and organizations. Connecting local interpretations on law to an international context also makes it easier to compare decisions and encourage the development of trust that is necessary for collaboration using sensitive genomic data. It is therefore advisable for biobank builders to adopt a system of governance where:

- The ethical standards set forth by the Global Alliance for Genomics and Health are upheld [5].
- Samples are stored and managed in accordance with the internationally recognized ISBER standards for best practice [41].
- Sharing is handled in a manner compliant with the International Charter of principles for sharing bio-specimens [42].

This does not preclude researchers from having to abide by the national law of each state involved in international research collaborations and is far from an exhaustive list of tools to support international sharing of samples. But it may provide an international research project with a common foundation and framework, which make the project more easily acceptable to the national authorities charged with reviewing projects.

The inherent adaptability of soft law also mean that international collaboration through soft law mechanisms may steadily improve, as experience is gained among stakeholders and thus alleviate the need for global governance via codified hard law solutions within the field. If given time to adapt, researchers and associated organizations might instead be able to contribute to a bottom-up harmonization of a soft global bioethical framework.

Key Points

- To accommodate international collaboration, it is necessary to bridge the gap between national legal frameworks. This is usually done by designated experts and organizations who determine if material transfer agreements are able to protect the rights of the donors in accordance with what they could expect when giving their consent for samples to be stored for future usage.
- Collaboration is substantially more likely to be accepted between nations where the respective authorities have had the possibility to become familiar with each other’s customs and traditions. Identifying successful precedents by other researchers participating in collaborative projects can therefore greatly reduce the time necessary to access samples.
- Different institutions define terms such as consent, informed consent and broad consent differently. This mean that an ‘informed consent’ at one institution may not be accepted as truly informed by another. Under such circumstances, researchers are likely to face a situation where the strictest interpretation in terms of data protection or privacy becomes the governing one.
- There is a conflict between reciprocity, anonymity and the right to not know. Research must therefore be planned and conducted in accordance with what the donors could reasonably expect when donating their samples and giving their consent.

## Funding

This work was financed by the BBMRI-LPC and the B3Africa projects. BBMRI-LPC is supported by the European Community's 7th Framework Programme (FP7/20072013) grant agreement no. 313010, B3Africa is supported by the European Union s Horizon 2020 research and innovation programme under grant agreement No 654404.

## Notes

**Tomas Klingström** is a PhD student at the Swedish University of Agricultural Sciences (SLU) and employed at SLU-Global Bioinformatics Centre. His main research deals with data integration and quality verification of the scientific process from sample procurement to data analysis working in the BBMRI-lpc, BBMRI.se and B3Africa projects.

**Erik Bongcam-Rudloff** is a professor in Bioinformatics at the Swedish University of Agricultural Sciences (SLU) and the head of the SLU-Global Bioinformatics Centre. His main research deals with the development of bioinformatics solutions for the Life Sciences community including nematode, plant, animal and human genomics research; numerous projects on metagenomics; and the building of LIMS and Biobank systems based on open-source elements (www.b3africa.org).

**Jane Reichel** is a professor in Administrative Law at the Faculty of Law and is tied part-time to the Centre for Research Ethics and Bioethics, both at Uppsala University. She is currently vice dean and chairman of the research committee at the Faculty of Law. Her research focuses on the globalization and Europeanization of administrative law, mainly in the areas of transparency and data protection in cross-border biomedical research.
